# Analysis of Weather Factors on Aircraft Cancellation using a Multilayer Complex Network

**DOI:** 10.3390/e25081209

**Published:** 2023-08-14

**Authors:** Kyunghun Kim, Hoyong Lee, Myungjin Lee, Young Hye Bae, Hung Soo Kim, Soojun Kim

**Affiliations:** 1 Department of Civil Engineering, INHA University, Incheon 22212, Republic of Korea; tgb611@naver.com (K.K.);; 2 Program in Smart City Engineering, INHA University, Incheon 22212, Republic of Korea; 3 Disaster Management Team, Department of Safety and Health, Korea Electric Power Corporation, Naju 58322, Republic of Korea; 4 Institute Water Resources System, INHA University, Incheon 22212, Republic of Korea

**Keywords:** aircraft cancellation, multilayer complex network (MCN), network analysis, centrality analysis

## Abstract

Airlines provide one of the most popular and important transportation services for passengers. While the importance of the airline industry is rising, flight cancellations are also increasing due to abnormal weather factors, such as rainfall and wind speed. Although previous studies on cancellations due to weather factors considered both aircraft and weather factors concurrently, the complex network studies only treated the aircraft factor with a single-layer network. Therefore, the aim of this study was to apply a multilayer complex network (MCN) method that incorporated three different factors, namely, aircraft, rainfall, and wind speed, to investigate aircraft cancellations at 14 airports in the Republic of Korea. The results showed that rainfall had a greater impact on aircraft cancellations compared with wind speed. To find out the most important node in the cancellation, we applied centrality analysis based on information entropy. According to the centrality analysis, Jeju Airport was identified as the most influential node since it has a high demand for aircraft. Also, we showed that characteristics and factors of aircraft cancellation should be appropriately defined by links in the MCN. Furthermore, we verified the applicability of the MCN method in the fields of aviation and meteorology. It is expected that the suggested methodology in this study can help to understand aircraft cancellation due to weather factors.

## 1. Introduction

Aircraft have become an important means of transportation. Prior to the COVID-19 pandemic (2010–2019), the number of customers and amount of cargo weight using aircraft had increased annually by 6.3% and 4.4%, respectively. According to the Organization for Economic Co-operation and Development (OECD) statistics, before the COVID-19 pandemic, the total number of international departures for tourism also showed 5% annual increases. One of the most important factors in aircraft operations is the weather [1]. The intensity and frequency of weather phenomena, such as heavy rainfall and high wind speed, are increasing due to climate change, with a corresponding adverse effect on the aviation industry. According to statistics from the Department of Transportation, USA (https://www.transportation.gov/, accessed on 7 August 2023), there were 78,214 aircraft cancellations before the COVID-19 pandemic due to weather. It also showed that more than half of the total cancellations were caused by weather each year. This also occurred in other regions such as Europe and Asia. In the Republic of Korea, the number of cancellations due to weather is increasing annually, accounting for more than 80% of the total [2]. Many experts have projected that the cancellations will be further exacerbated by the ongoing climate change [3,4]. 

There are many studies on aircraft cancellation due to weather. Sasse and Haurf [5] were the first to quantify the impact of thunderstorms on landing aircraft at Frankfurt Airport in Germany. Park et al. [6] indirectly estimated the economic impact of flight cancellations due to fog at Incheon International Airport. Lee et al. [7] conducted a statistical analysis of flight cancellations and delays at domestic airports, finding that each airport was impacted differently by meteorological factors. Shultz et al. [8] evaluated the impact of weather events on airport performance and selected appropriate thresholds for significant weather conditions. Alexander and Onyejiri [9] investigated the effect of adverse weather on air transport at Port Harcourt international airport, showing that it affects aircraft operation, passenger finances, and health. Lee et al. [10] projected the number of flight cancellations and corresponding economic losses based on climate change scenarios. These studies show that aircraft cancellations are a phenomenon caused by weather factors affecting aircraft operations, and weather and aircraft should be considered concurrently in research. In this context, a multilayer complex network method could be a useful tool to investigate cancellations.

The complex network method simplifies a target or phenomenon visually into a graph or network and derives useful information, such as features of the target and an understanding of the physical behaviors, roles, and interactions between components and their relationships [11,12]. In addition, it has high applicability to a wide range of systems and phenomena. Despite the diversity of networks found in nature and society, many of them share common underlying principles and structures, such as small-world and scale-free networks [12,13]. This similarity enables researchers to use the same set of mathematical and computational tools to explore and analyze various networks. In transport infrastructures, the complex network method has been widely applied to areas such as traffic [12,13,14,15], railways [16,17,18,19], and especially the air transport system [20,21,22,23]. Previous studies unveiled the topological structure and dynamic behavior of the air transport network, where airports are denoted by nodes and flights between airports are denoted by links. 

However, as a system or phenomenon gradually becomes more complex, limitations begin to appear when it is analyzed as a single network. To solve this problem, researchers began to define systems using a multilayer network called a “Multilayer Complex Network (MCN)”. In an MCN, several layers are built for each factor of the system, and connections are then defined between the layers through the relationships between the factors to construct a multilayer network. An MCN has the advantages of a complex network, as well as other advantages [24]. Compared with a single-layer network, multilayer networks incorporate diverse nodes and multiple types of links in network elements, allowing for a more comprehensive characterization of the complex components and their correlations. In addition, the multilayer structure provides an analytical approach to explore layer interactions, which enables the analysis of topological properties that are significantly different from each single layer [25]. The concepts related to MCNs were introduced in the study of air transport networks [26,27,28,29,30,31,32]. These studies constructed MCNs of air transport by using aircraft factors, like routes between airports, the flight schedule, and the number of aircraft seats. After making the MCN, they applied several network analyses to find out the characteristics of the MCN, like robustness, aggregation, and complexity. Cardillo et al. [26] constructed the European air transport network where the 15 biggest airline companies in Europe were considered as 15 layers. These found that the multilayer structure strongly reduces the robustness of the system. Du et al. [28] established the Chinese airline network as multilayer infrastructure via the k-core decomposition. The k-core decomposition divides nodes in a single-layer network into several layers according to the number of node connections to make the MCN. It allowed for easy identification of characteristics of the network compared with a single-layer network. Gaggero and Piazza [29] applied multilayer network theory to the US domestic market observed during the period 2012–2019 and analyzed the effect of the airline network on the carriers’ decision to open a new route. Also, they applied dynamic models to investigate the tendency of expansion of an aircraft route. Ren et al. [30] combined the air sector network with the aircraft state network to create the aircraft control multilayer network. It showed that the multilayer-based method is effective and reliable for the robustness analysis. Although previous studies only considered aircraft factors in a multilayer complex network method, this study attempted to analyze aircraft by considering both aircraft and weather factors. 

This study analyzed aircraft cancellations due to weather in the Republic of Korea using the MCN method. We constructed a multilayer network using three distinct layers: rainfall, wind speed, and aircraft. The aircraft layer was based on the schedule data of each airport, while the other layers utilized weather data from the Automated Synoptic Observing System (ASOS) near the airports. To establish connections between the layers, we used the number of aircraft cancellations attributed to each weather factor because aircraft cancellation due to weather is an event where weather directly affects the operation of aircraft. After the construction, we analyzed the MCN with several network analyses (global degree distribution and strength distribution, rich-club coefficient, clustering coefficient, and network assortativity coefficient) and identified characteristics of the cancellations using the network analysis results. In addition, we looked at which airports had the most important role in the network by applying adjacency information entropy, which is one of the centrality measures. Lastly, the importance of network construction considering the purpose and characteristics of this research is provided. 

The remainder of this paper is organized as follows. Section 2 introduces the study area and research data. Section 3 presents the research methodologies used in the study. The analysis results and discussions are in Section 4. Finally, conclusions are provided in Section 5. 

## 2. Methods and Materials

### 2.1. Aircraft Cancellation Criteria

The Korea Aviation Meteorological Agency (KAMA) manages aircraft safety by issuing aeronautical meteorological warnings when weather, which may adversely affect aircraft on the ground (including parked aircraft, aerodrome facilities, and services) is observed or predicted [33]. The warnings reflect specific types of weather, as shown in the Table 1.

### 2.2. Complex Network

A complex network is based on basic network analysis, and the word “Complex” is added to reflect the increased complexity in the amounts and forms of data [34]. The first step in applying a complex network is to define the “nodes” and “links”, which are the basic factors of a network. A node is an entity within an analysis target and represents an intersection in a network. The links are connections between nodes. For example, in a subway, each subway station is a node, and the railways between the stations are the links. The most influential factor in the complex network is the link. The type of link defines the type of network. Depending on the presence or absence of the direction and weight of the link, a network can become directed/undirected or weighted/unweighted. From a mathematical point of view, we can present a network by means of an adjacency matrix. If there are *N* nodes in a network, the matrix has an *N* × *N* shape. The links in the network can be represented by the elements $A_{ij}$ of the *matrix*:

(1) $$A_{ij}=\left\{\begin{array}{c} w_{ij},\;\mathrm{if}\;\mathrm{the}\;\mathrm{nodes}\;i\;\mathrm{and}\;j\;\mathrm{are}\;\mathrm{connected}\;\mathrm{with}\;\mathrm{weight}\\ 0,\;\mathrm{otherwise} \end{array}\right.$$

Generally, $w_{ij}$ has a value between 0 and 1.

It is easy to define links in a network where there is a clear connection, such as a road or aircraft. In aircraft-related complex network studies, they used the number of aircraft between airports or passengers for calculating the weights of links [20,21,22,23,26,27,28,29,30,31,32]. However, if there is no clear connection, the researcher must confirm the connectivity between nodes. The correlation method is widely used to check the connectivity but encompasses some uncertainty because it relies on the researcher’s judgment in choosing a threshold for the coefficient to determine whether there is a connection [35]. Event synchronization is a method used to overcome the uncertainty of the correlation method [36]. Instead of relying on a threshold for the correlation coefficient, it calculates the degree of synchronization between two points based on the number of events that occur within a specific period. It is useful for constructing networks in which the connectivity between nodes is not immediately apparent. The procedure for calculating the event synchronization is as follows [37]:

1. Determine the threshold value for a time series of nodes, which represents the standard at which the target event occurs.
2. At two different nodes *A* and *B*, calculate the occurrence time of the target events for each point, and then estimate the time intervals between the events. Select the shortest interval ($T^{AB}$) as the time interval of the target events for calculating event synchronization.
3. Identify the events in node *B* that occur within $T^{AB}$ of the events at node *A*. If the events occur simultaneously at nodes *A* and *B*, they are assigned a weight of 0.5.
  (2) $$c\left(x|y\right)=\sum_{x=1}^{s_{A}}\sum_{y=1}^{s_{B}}J_{xy}$$
  (3) $$J=\left\{\begin{array}{c} 1.0\;\;if\;0<t_{x}^{A}-t_{y}^{B}<T^{AB}\\ 0.5\;\;if\;t_{x}^{A}=t_{y}^{B}\\ 0.0\;\;otherwise \end{array}\right.$$
  where $s_{A}$ and $s_{B}$ are the number of target events at nodes *A* and *B*, respectively, while $t_{x}^{A}$ and $t_{y}^{B}$ represent the occurrence time of the *x*^th^ target event at node A and the *y*^th^ event at node *B*.
4. Calculate the event synchronization value between nodes *A* and *B*:
  (4) $$Q_{AB}=\frac{c\left(x|y\right)+c\left(y|x\right)}{\sqrt{\left(s_{A}-2)(s_{B}-2\right)}}$$
  The range of the event synchronization is from 0 to 1. If the value is 1, the two nodes are perfectly synchronized. If it is 0, it is interpreted as no correlation because no common events are shared.

To construct the three different layers, event synchronization is applied to calculate the weights of links in the rainfall and wind speed layers. Regarding the aircraft layer, we calculated the weights of links based on the number of aircraft operations from 2009 to 2021 provided by the Korea Airports Corporation (KAC). For example, let us consider three other airports (B, C, and D) that operate flights to airport A. Suppose these airports have 20, 30, and 50 flights to airport A, respectively. In this case, we calculate the weight of the link from airport B to airport A ($w_{BA}$) as 0.2 (i.e., $\frac{20}{20+30+50}$).

### 2.3. Multilayer Complex Network Analysis

**Figure 1:**
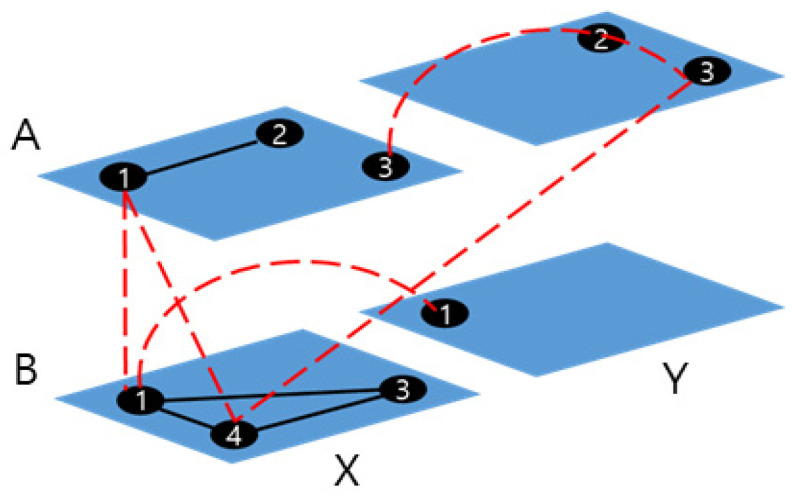
Shape of a multilayer network.

In contrast with the past, where the factors were often characterized by single elements, contemporary events often involve complex interactions between factors with different characteristics. As a result, it may be difficult to represent such events using a single-layer network. The multilayer concept has been applied to address these limitations [24]. A multilayer network is composed of multiple single layers, each with its own set of nodes and links. The network contains two types of links: intra-layer links that connect nodes within the same layer and inter-layer links that connect nodes across different layers. Figure 1 depicts a multilayer network composed of four layers (*AX*, *AY*, *BX*, and *BY*). In each layer, black lines represent intra-layer links, while red lines denote inter-layer links. Intra-layer links are links inside of a layer and inter-layer links cross the layers.

Two types of links enable the representation of complex phenomena and relationships by incorporating various factors. However, as the network grows, and thus, the complexity with a diverse set of elements, the number of calculations required increases exponentially. Despite this challenge, multilayer complex networks are widely used in various fields, including genetics, neurology, and sociology, as they can provide valuable insights into phenomena. In addition, the MCN fits the real situation more suitably, as it can accurately define how the different dynamics develop in each layer of a complex system [28].

By using the definition of aircraft cancellation due to weather, we calculated the weights of the inter-layer links between the aircraft layer and two weather layers based on aircraft cancellation data. For example, if there are 20 and 30 aircraft cancellations due to rainfall and wind speed, respectively, the weights of inter-layer links are 0.4 (i.e., $\frac{20}{20+30}$) and 0.6 (i.e., $\frac{30}{20+30}$). However, we did not consider inter-layer links between the rainfall and wind speed layers despite several past studies that have shown that wind speed and rainfall are correlated; this will be explained in Section 3.3 with the importance of proper construction of the multilayer complex network.

**Figure 2:**
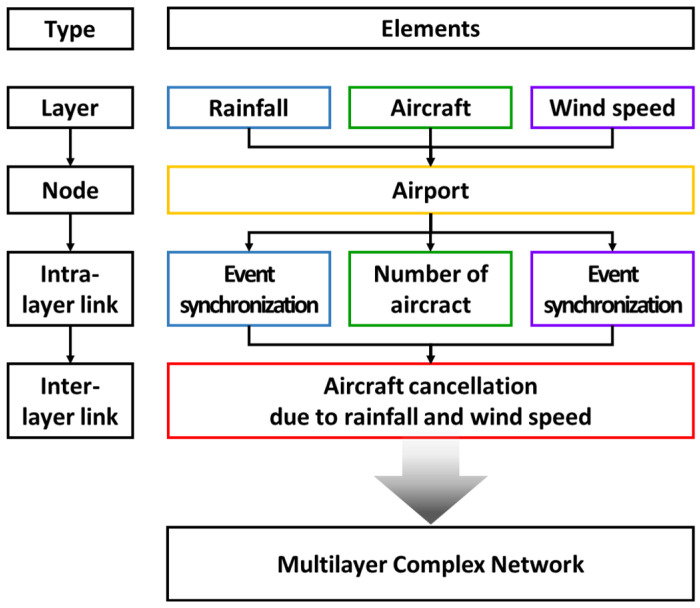
Process of constructing a multilayer complex network.

**Figure 3:**
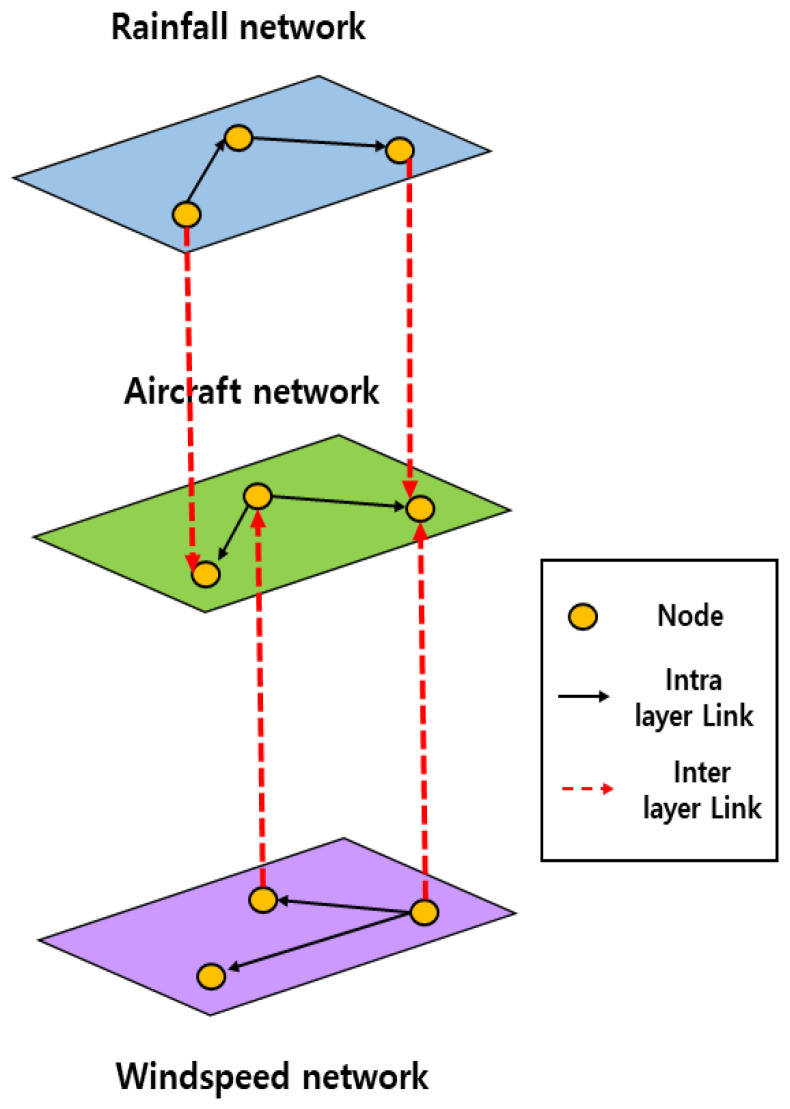
Conceptual structure of multilayer complex network.

The entire process of constructing the MCN is the same as shown in Figure 2, and the conceptual structure of the network is depicted in Figure 3.

### 2.4. Network Analysis

After constructing the MCN, we applied five network analyses: degree distribution, rich-club coefficient, clustering coefficient, network assortativity coefficient, and centrality analysis. The subsections below explain each analysis method.

#### 2.4.1. Degree Distribution

Each node in a network has a different number of links, which is called its “degree”. In the case of a weighted network, links have their own weights, and the total weight of all links connected to a node is referred to as its “strength”. The degree of the nodes can be expressed as a probability density function, which is known as the degree distribution. The degree distribution represents the probability (p(k)) that any node has a degree value of k. For example, if all nodes have two links, then p(k = 2) is 1. The strength distribution uses a similar concept to the degree distribution, but instead of looking at the degree, it considers the strength. In other words, the strength distribution represents the probability (p(s)) that any node has a total link weight of s. By analyzing the degree and strength distributions, researchers can gain insights into the distribution of links and the overall structure of the network [38]. 

#### 2.4.2. Rich-Club Coefficient

The rich-club coefficient is an index used to quantify the level of connectivity between hub nodes in a network [39]. Hub nodes are those with a high number of links. The rich-club coefficient is calculated by dividing the number of links between hub nodes by the maximum possible number of links between them (Equation (5)).

(5) $$\emptyset \left(k\right)=\frac{2E_{\geq k}}{N_{\geq k}(N_{\geq k}-1)}$$

where $E_{\geq k}$ represents the number of links between nodes with a degree greater than or equal to *k*, while $N_{\geq k}$ is the number of nodes with a degree greater than or equal to *k*. 

A higher value of the rich-club coefficient indicates that hub nodes in the network share a greater number of links with each other. This can be interpreted as evidence of a more tightly knit and cohesive structure within the network. An example of a network with a high rich-club coefficient is a power grid network. Power grids are designed to provide backup and supplementary power to major facilities in case of emergencies or outages. The high level of interconnectivity between hub nodes in the power grid ensures that the system can withstand external shocks and continue functioning even in the event of failures at critical nodes [40]. 

#### 2.4.3. Clustering Coefficient

The clustering coefficient is a key index used in the analysis of aircraft networks [41]. The clustering coefficient measures the extent to which nodes in a network tend to cluster together and form tightly interconnected subgroups or communities. In aircraft networks, high clustering coefficients indicate the presence of a closely connected group of airports or airlines that share common routes, passengers, or operational resources. In a general network, the clustering coefficient represents the degree of clustering between nodes. The coefficient for a node *i* is calculated as follows:

(6) $$C\left(v_{i}\right)=\frac{\left|\left\{\left(v_{x},v_{y}\right)|A_{ix},A_{iy},A_{xy},x\neq y\right\}\right|}{\left|\left\{(v_{x},v_{y}|A_{ix},A_{iy},x\neq y\right\}\right|}$$

where $A_{xy}$ denotes the connection between node *x* and node *y*.

It yields a value between 0 and 1, where a higher value indicates a greater degree of clustering tendency for nodes. In the case of a weighted network, the clustering coefficient is calculated by considering the weights of the links:

(7) $$C\left(v_{i}\right)=\frac{\left|\left\{\left(v_{x},v_{y}\right)|W_{ix},W_{iy},W_{xy},x\neq y\right\}\right|}{\left|\left\{(v_{x},v_{y}|W_{ix},W_{iy},x\neq y\right\}\right|}$$

where $W_{xy}$ denotes a link weight between node x and node y.

The clustering coefficient can be calculated for individual nodes, as well as for the network. The local clustering coefficient measures the degree to which the neighbors of a particular node are connected. The global clustering coefficient is the average of the local clustering coefficient for all nodes in the network. A network with a global clustering coefficient close to zero indicates that the connections between nodes are mostly random, while a high global clustering coefficient suggests strong connections between nodes, indicating a more organized and structured network [42].

#### 2.4.4. Network Assortativity Coefficient

The network assortativity coefficient measures the extent to which nodes with similar characteristics (degree, strength, etc.) tend to be connected. It is calculated as follows:

(8) $$r=\frac{M^{-1}\sum_{i}j_{i}k_{i}-{\left[M^{-1}\sum_{i}\frac{1}{2}(j_{i}+k_{i})\right]}^{2}}{M^{-1}\sum_{i}(j_{i}^{2}+k_{i}^{2})-{\left[M^{-1}\sum_{i}\frac{1}{2}(j_{i}+k_{i})\right]}^{2}}$$

where $j_{i}$ and $k_{i}$ are the degrees of the nodes located at the end of the ith link, and *M* is the total number of links in the network. 

The network assortativity coefficient ranges from −1 to 1, where a positive value indicates an assortative network and a negative value indicates a disassortative network. An assortative network tends to aggregate nodes with similar characteristics, resulting in high efficiency, strength, and stability due to the condensed nature of its nodes. On the other hand, a disassortative network tends to connect nodes with different characteristics, which can lead to increased resilience and robustness [43].

#### 2.4.5. Centrality Analysis

The nodes in a network have different levels of importance according to their location or number of links. Among the nodes, those with high importance have a significant influence on the structure or function of the network. Identifying these nodes in a network is important for both theory and practice [44]. For example, if the government identifies the transformers playing key roles in a power grid, it can prepare investment or defense measures for them in advance. Centrality analysis is a methodology used for calculating the importance of nodes. Various methodologies have been developed for centrality analysis, including degree centrality, betweenness centrality, closure centrality, eigenvector centrality, and page rank centrality [45]. Among the various methods, this study calculated the centrality of nodes in the multilayer complex network through adjacency information entropy. The method was proposed by Xu et al. [44] and utilizes information entropy for its calculations. The method is applicable to all types of networks and has shown results in identifying critical nodes more accurately compared with existing methods. The calculation procedure of adjacency information entropy is as follows:

1. Calculate an adjacency degree ($A_{i}$). The adjacency degree considers the nearest neighbor nodes.
  (9) $$A_{i}=\sum_{j\in \Gamma_{i}}k_{j}$$
  where *j* is the neighbor of node *i*, $\Gamma_{i}$ is the set of neighbors of node *i*, and $k_{j}$ is the degree of node *j*. If a network is weighted, $k_{j}$ is changed into strength $w_{j}$.
2. Calculate a selection probability ($P_{i_{j}}$). From the viewpoint of information theory, a certain node in a network takes charge of the information source point, and its neighboring nodes are taken as the target points. In the process of information transmission, the source point will select a target point among its neighboring nodes for transmission. The probability that the target nodes are selected is the selection probability. It considers the importance of the selected nodes.
  (10) $$P_{i_{j}}=k_{j}/A_{j},\;(j\in \Gamma_{i})$$
3. Calculate an adjacency information entropy ($E_{i}$). The adjacency information entropy shows how much importance each node has in the network.
  (11) $$E_{i}=-\sum_{j\in \Gamma_{j}}\left(P_{i_{j}}{log}_{2}P_{i_{j}}\right)$$
  The larger the entropy value of a node, the more important the role the node plays in the network.

### 2.5. Study Area and Data Collection

**Figure 4:**
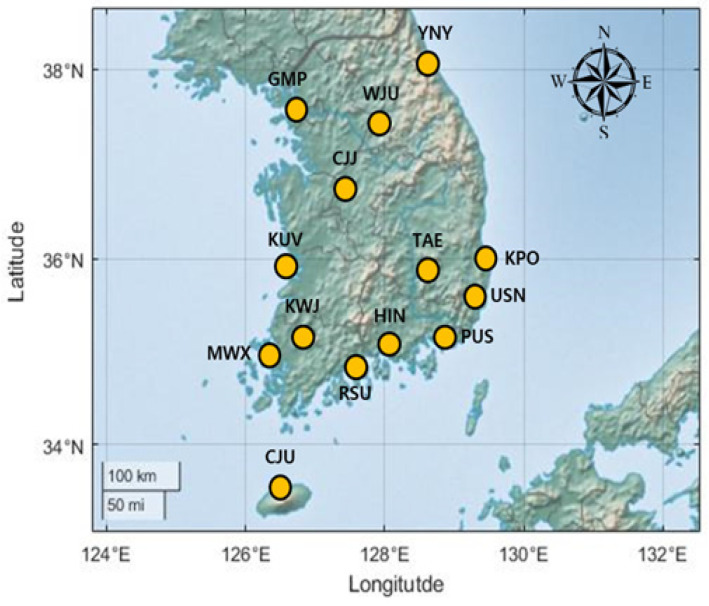
Location of the 14 studied airports in the Republic of Korea.

This study constructed the MCN for 14 airports located in the Republic of Korea (Figure 4): Gwangju (KWJ), Gunsan (KUV), Daegu (TAE), Muan (MWX), Gimhae (PUS), Gimpo (GMP), Yangyang (YNY), Yeosu (RSU), Ulsan (USN), Wonju (WJU), Jeju (CJU), Sacheon (HIN), Cheongju (CJJ), and Pohang (KPO). We excluded Incheon international airport because there are not many domestic air routes to other airports. Most of the airports are in the southern part of the Korean Peninsula (Figure 4). We collected flight schedule data from Air Portal (https://www.airportal.go.kr, accessed on 19 June 2023), which is managed by the Ministry of Land, Infrastructure, and Transport, Republic of Korea, covering the period of 2009–2021. The portal provided the schedule data from 2009; this data showed that the number of scheduled flights decreased because of the COVID-19 pandemic from the end of 2021. Daily rainfall and maximum wind speed data were also collected from ASOS stations near the airports. We attempted to use meteorological data from the airports, but some airports did not provide data for the period between 2009 and 2021, and there were many missing data. The meteorological data from ASOS stations are available on the Open MET Data Portal (https://data.kma.go.kr, accessed on 19 June 2023) managed by the Korea Meteorological Administration. 

## 3. Results

### 3.1. Construction of the Multilayer Complex Network

**Figure 5:**
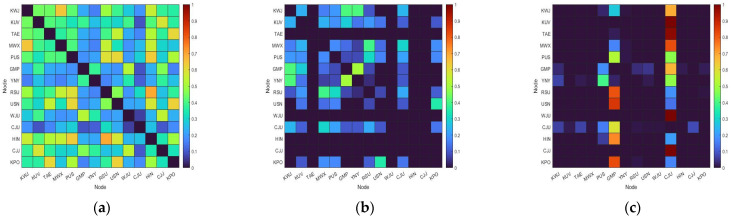
Adjacency matrices of the three single networks: (**a**) rainfall; (**b**) wind speed; (**c**) aircraft. Red represents a high weight, while blue indicates a low weight.

As explained in Section 2.2, we constructed single-layer networks for rainfall, wind speed, and aircraft. First, we calculated the adjacency matrices (Figure 5). The matrices for rainfall and wind speed show that they had symmetrical shapes. 

This is because the same value was calculated when the same two nodes were reversed. In contrast, an asymmetric form occurred in the aircraft network because the number of flights between each pair of airports varied. Unlike the adjacency matrix of the rainfall network, the others had a lot of zero values. Rainfall is a weather phenomenon that occurs widely. Specifically, during the rainy season, more than 30% of the total annual precipitation occurs throughout the entire Korean Peninsula, allowing for each region to experience many of the same rainfall events. On the other hand, wind speed is a local weather phenomenon, and thus, the degree of synchronization with other areas is low. In the case of aircraft, there are 60 air routes between 14 airports, with 44 of them arriving or departing from GMP and CJU. This characteristic is reflected in the aircraft adjacency matrix.

**Figure 6:**
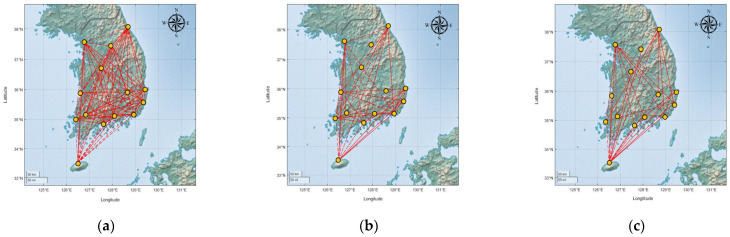
Three single-layer networks: (**a**) rainfall; (**b**) wind speed; (**c**) aircraft. The yellow dots represent nodes and the red dotted lines denote links. According to the adjacency matrices, the rainfall network had the highest number of links. Additionally, in the aircraft network, most of the links were connected to the GMP and CJU nodes.

Figure 6 displays each single-layer network. By comparing the number of links in the three networks, we can observe that rainfall had an overwhelmingly large number of links (182), while wind speed and aircraft networks had similar numbers (wind speed: 78, aircraft: 60). The average weights of the links followed the order of rainfall (0.341), aircraft (0.233), and wind speed (0.188) networks. There was a significant difference between the rainfall and wind speed due to the large gap in the number of events that met the criteria for aircraft cancellation. Table 2 presents the number of events, and except for RSU and MWX, the number of rainfall events that exceeded 30 mm/h was much higher than those that exceeded 25 kt. As a result, the weights of the links in the wind speed network were much smaller than those in the rainfall network. 

**Figure 7:**
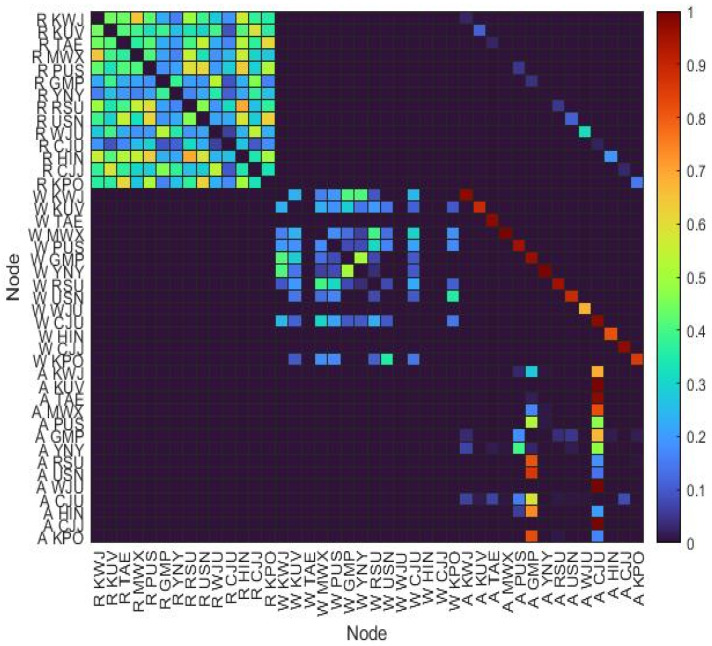
Adjacency matrix of the MCN: R indicates the rainfall network, W represents the wind speed network, and A is the aircraft network. The adjacency matrix of the MCN contains all matrixes of the single-layer networks.

**Figure 8:**
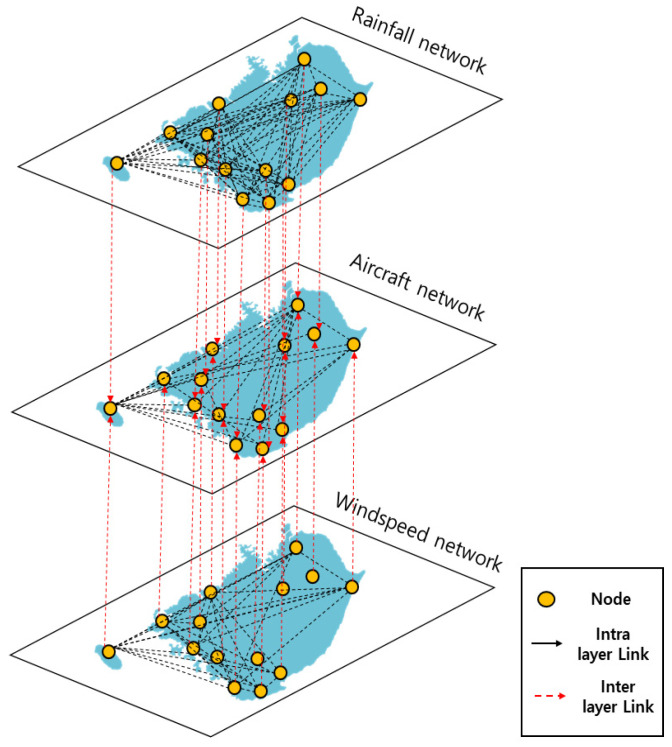
Construction of the multilayer complex network: the yellow dots are nodes, the black dotted lines are intra-layer links, and the red dotted lines are inter-layer links.

The multilayer network was constructed from the single networks using aircraft cancellation data. By examining the adjacency matrix of the multilayer complex network (Figure 7), as opposed to the rainfall network, it is apparent that the wind speed network had strong inter-layer link weights with the aircraft network. This was because the number of cancellations due to wind speed was significantly larger. The structure of the multilayer network is shown in Figure 8. The total number of links was 346 (intra-layer links: 320, inter-layer links: 26). In the case of MWX and YNY, aircraft cancellations were caused only by wind speed, and thus, only the inter-layer link between the wind speed and aircraft existed.

### 3.2. Network Analysis of the Multilayer Complex Network

In this section, we analyzed the MCN using the methods introduced in Section 2.4. 

#### 3.2.1. Degree and Strength Distribution

**Figure 9:**
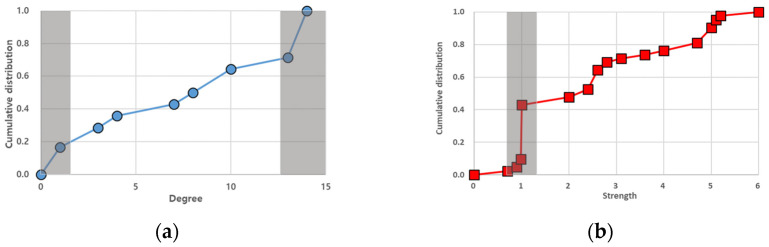
Node distribution of the MCN: (**a**) degree distribution; (**b**) strength distribution. The gray-shaded regions represent the ranges that show a higher slope.

**Figure 10:**
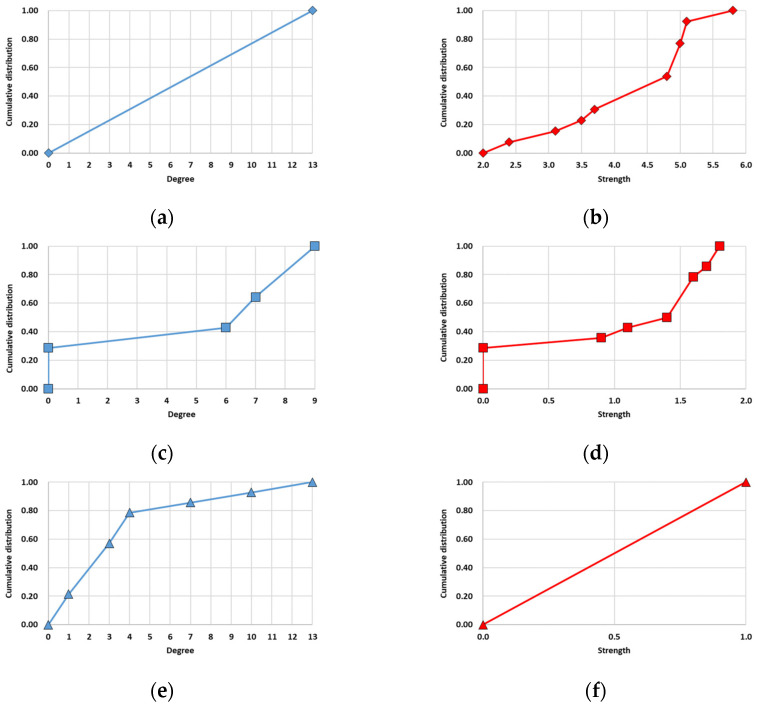
Node distribution of the single networks: (**a**) degree distribution of the rainfall network; (**b**) strength distribution of the rainfall network; (**c**) degree distribution of the wind speed network; (**d**) strength distribution of the wind speed network; (**e**) degree distribution of the aircraft network; (**f**) strength distribution of the aircraft network. Each single-layer network had a different shape of distribution because of each data characteristic.

We analyzed the structure of the single-layer networks and multilayer networks by considering the degree and strength distribution (Figure 9 and Figure 10). The degree distribution of the multilayer network shows that the slope was higher at the beginning and end than in the rest, indicating that the distribution of the number of links in each node was concentrated at both ends. In the strength distribution that considered the weights of links, a higher slope occurred only once. This indicated that there was a higher concentration of nodes with a strength value near one. Apart from this range, the strength of other nodes was almost distributed equally.

The results for the degree and strength distributions of the single-layer networks were the same as those in Figure 10. All the nodes in the rainfall network were the same degree of 13. In the case of strength, many nodes had a value of 4.8 or higher. Nodes with no links existed in the wind speed network, and they were mostly located in inland areas, like TAE, WJU, HIN, and CJJ. The aircraft network had many nodes with four or fewer links. Additionally, all the nodes had the same strength of one because of the link weight calculation method used in the aircraft network.

A comparison of the results revealed that the characteristics of the single-layer networks were reflected in the multilayer complex network. In the degree distribution of the multilayer complex network, the initial sharp slope was caused by nodes with a low number of links in the wind speed and aircraft networks, while the latter originated from nodes in the rainfall network with many links. In the strength distribution of the multilayer complex network, the sharp slope was caused by the nodes in the aircraft network having the same strength value (=1.0).

#### 3.2.2. Rich-Club Coefficient

To identify hub nodes in the MCN, we utilized the rich-club coefficient. The range of the number of links was set from 1 to 14 because the maximum degree was 14.

**Figure 11:**
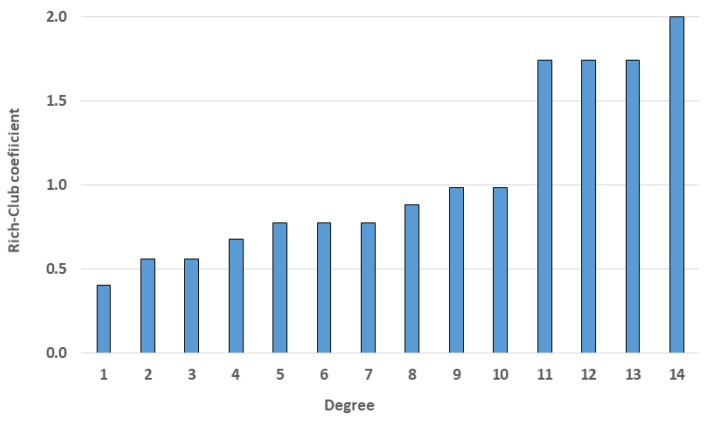
Rich-club coefficient result. From the degree of 11, there was a rapid increase in the rich-club coefficient.

As illustrated in Figure 11, the coefficient value increased as the degree of the node increased. In addition, there was a sharp increase in the degree from 11 onward. Based on this characteristic, we defined those nodes with over 11 links as hub nodes. With respect to the locations of the hub nodes, all hub nodes, except for the CJU in the aircraft network, were in the rainfall network. This means that nodes with a high degree were concentrated in a certain layer. Based on the results of the rich-club coefficient analysis, we concluded that the hub nodes of the multilayer complex network had a tightly knit and cohesive structure. In addition, they were concentrated within a specific layer of the network.

#### 3.2.3. Clustering Coefficient

**Figure 12:**
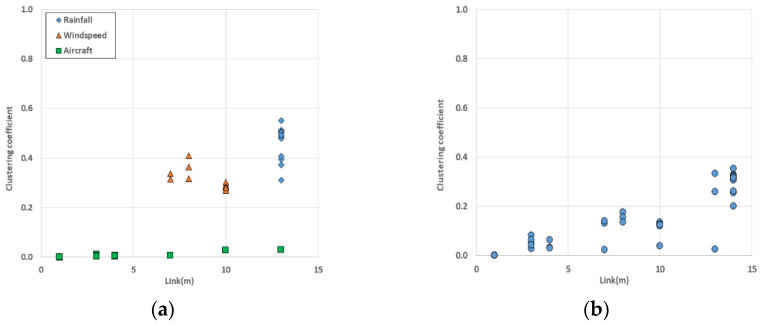
Clustering coefficient result: (**a**) single-layer networks (diamond: the rainfall network, triangle: the wind speed network, square: the aircraft network); (**b**) MCN. Both results show that the networks had weak connections between the nodes.

The clustering coefficient helps to identify connectivity between nodes in the network. We calculated local clustering coefficients of nodes in the single-layer networks and the multilayer network (Figure 12). 

When looking at the result of aircraft single layer network (Figure 12a), three nodes (CJU, GMP, and PUS) had higher value of clustering coefficient because they had more links than the other nodes. The global clustering coefficient of the wind speed network was 0.227, and the local clustering coefficients of four nodes (TAE, WJU, HIN, and CJJ) were 0. This was because these nodes only had one link in the network. Among the three single-layer networks, the rainfall network stood out with the highest global clustering coefficient of 0.465, indicating a high level of connectivity between the nodes. This could be attributed to the significantly higher number of links present in the rainfall network compared with the other single networks. The MCN had a global clustering coefficient of 0.144. When comparing the local clustering coefficients of nodes in the multilayer complex network and single-layer networks, an increase was observed only in the local clustering coefficient of nodes located in the aircraft network. The calculated global and local clustering coefficients indicated that the multilayer complex network had less connectivity between the nodes. Including the result of the rich-club coefficient, we concluded that the MCN generally exhibited loose connectivity between the nodes; however, the hub nodes within the network were attached with strong bonds. 

#### 3.2.4. Network Assortativity Coefficient

The value of the network assortativity coefficient indicates a tendency to cluster nodes with similar characteristics. We calculated the coefficient into two cases, with and without weight, and obtained 0.628 and 0.428, respectively. Therefore, the MCN was an assortative network because both values were greater than 0. An assortative network tends to have nodes with a high degree or strength that prefer to connect with other nodes with a high degree or strength, while nodes with a low degree or strength tend to connect with other nodes with a low degree or strength. Therefore, it has high resilience and robustness to noise from outside. We had the same result from the rich-club and clustering coefficients. 

### 3.3. Centrality Analysis

We calculated the centrality of nodes using adjacency information entropy (Table 3 and Table 4). The nodes with higher centrality were considered more important or influential in the network. Table 3 represents the centrality analysis result of each single-layer network and Table 4 shows the results from the MCN.

**Figure 13:**
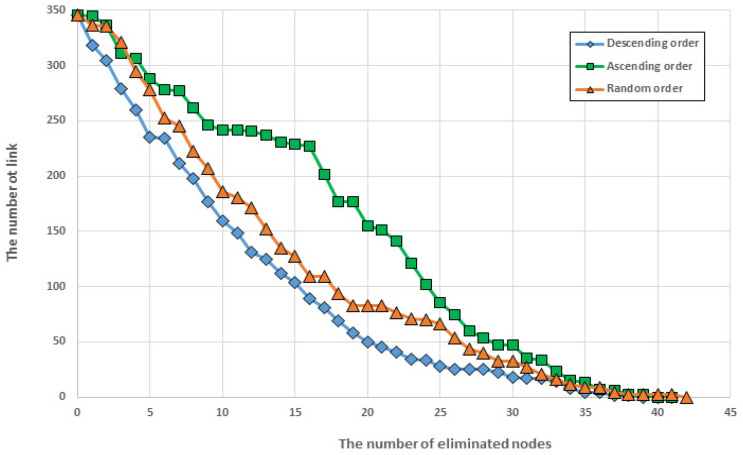
Change in the number links: descending order (diamond); ascending order (rectangular); random order (triangle). The most dramatic changes in the number of links are shown in descending order, while those shown in ascending order represent the least change.

To confirm whether the ranking based on the calculated entropy was correctly selected in the MCN, we examined the change in the number of links that appeared when the nodes were removed one by one. The node removal order was divided into three categories: removal in accordance with the calculated importance order (descending order), removal from the lower ranking (ascending order), and random selection. The calculated results (Figure 13) showed that removing nodes according to their importance ranking resulted in the sharpest change in the number of links, while removing nodes from the lower ranking resulted in the most gradual change. The result of randomly selecting nodes showed a change amount that fell between the two previous cases. The results confirmed that the calculated ranking was appropriate.

In the results of each single-layer network (Table 3), each network shows different results regarding the ranks. In the rainfall network, the KUV node was the most important node. Compared with the other networks, the entropy values of the nodes show smaller differences with each other. Regarding the wind speed network, nodes located near the coast had high ranks. In the aircraft network, nodes with high demand had high entropy values. 

Looking at the layers in the results of the MCN (Table 4), rainfall had the highest average rank of nodes (15.286) compared with the other two layers (wind: 22.071, aircraft: 27.143). In the rich-club coefficient result, we found that the rainfall layer had a higher number of hub nodes. Therefore, there was a high possibility that high-rank nodes were concentrated in the rainfall layer. When looking at the major rankings of individual nodes beyond the top five, the first and second nodes were occupied by CJU and GMP nodes in the aircraft layer, respectively. Third place belonged to WJU in the rainfall layer, followed by MWX in the wind speed layer at fourth place and USN in the rainfall layer at fifth place. All these nodes shared a common characteristic known as hub nodes. In contrast to the result observed in the rainfall layer, the most important node was in the aircraft layer. The CJU node in the aircraft layer had the highest number of links and the greatest strength among all nodes in the network. The reason why the CJU node exhibited these characteristics was due to the social and meteorological characteristics of CJU Airport. Jeju Island is located off the coast of the Korean Peninsula, and it has high tourism demand in the Republic of Korea. Most people travel to Jeju Island by aircraft, and as a result, not only airports around large cities (such as GMP and PUS) but also regional airports (such as WJU and YNY) have routes to this island. From 2009 to 2021, the number of aircraft that departed from or headed to CJU accounted for approximately 77% of the total domestic aircraft. Jeju Island has different characteristics in terms of meteorology. It is about 268 km away from Busan and in a transition zone between the subtropical and temperate climate zones. In addition, it is situated in the northwest Pacific Ocean, far from the Asian continent, and is affected by the humid ocean. Due to its location, Jeju Island experiences higher temperatures, more rainfall, and stronger winds than the Korean Peninsula. Therefore, compared with the airports located in the Korean Peninsula, CJU experiences a higher frequency of weather conditions that match the criteria of aeronautical meteorological warnings, resulting in numerous aircraft cancellations. 

In the results of the network analyses, we found that the characteristics of each single-layer network and inter-layer links affected the MCN. This feature can also be observed in the centrality analysis. Except for the CJU and GMP nodes in the aircraft layer, the others with a high rank in the MCN had lower importance in each layer. However, there was a common characteristic among the nodes, including the CJU and GMP nodes, in the aircraft layer, which was they all had inter-layer links with significant weights. Therefore, while the degree of importance that each node held within its respective layer was essential, it was also evident that the influence of inter-layer links was significant. Here, the weight of the inter-layer link represented a quantitative index that demonstrated how much each meteorological factor affected aircraft operations. 

## 4. Discussion

This study analyzed aircraft cancellations caused by weather factors using the MCN method. While building the multilayer complex network, we considered the relationship between the weather and aircraft layers but did not account for the relationship between different weather factors. To examine the relationship between rainfall and wind speed at each node, we calculated the correlation between the two factors, except for four airports (TAE, WJU, HIN, and CJJ) (Table 5); since those nodes did not experience any wind speed events that exceeded the aircraft cancellation criterion, they were excluded from the correlation analysis. In the calculated result, except for CJU and KPO, the other nodes did not exhibit significant correlation values.

**Figure 14:**
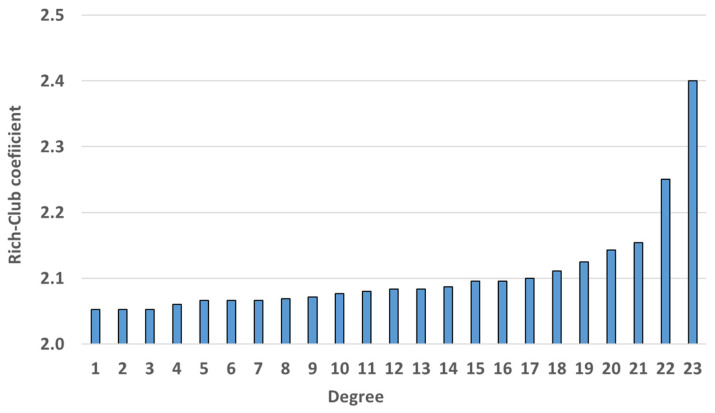
Rich-club coefficient results of the new MCN; from the degree of 22, there was a rapid increase in the rich-club coefficient.

Although we did not find a significant relationship between rainfall and wind speed, we attempted to add inter-layer links between the rainfall and wind speed layers to examine changes in the characteristics of the network. The new multilayer complex network had a total of 117 additional inter-layer links compared with the original one. There were changes in all the network analysis results, especially in the rich-club coefficient, clustering coefficient, and centrality analysis, which show significant differences. In the rich-club coefficient result (Figure 14), all coefficients of nodes were bigger than two. In addition, there was a sharp increase in the degree from 22. Therefore, in the new network, nodes with more than 22 links were defined as hub nodes. We compared the hub nodes in the original and new networks and found that the hub nodes of the new network were in the rainfall and wind speed layers, while those in the original network were in the rainfall and aircraft layers. 

**Figure 15:**
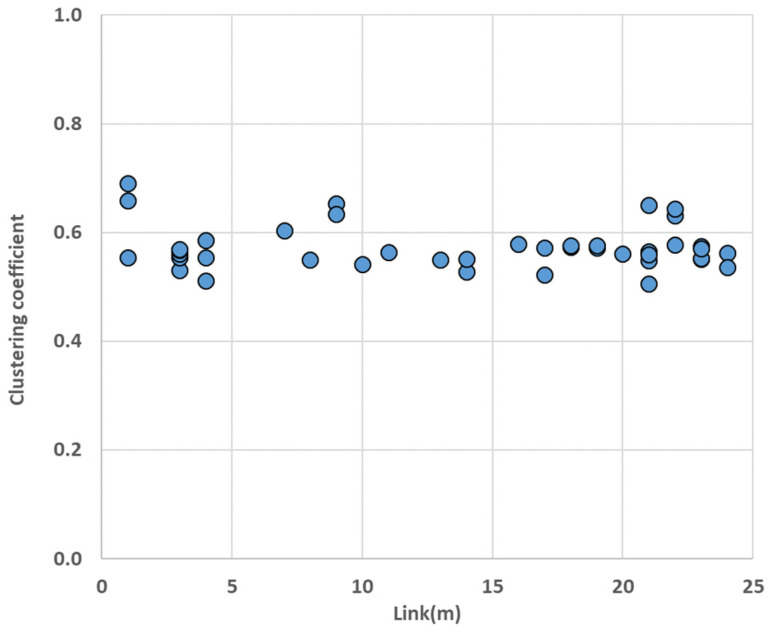
Clustering coefficient of the new MCN; compared with the coefficient of nodes in the original MCN, all coefficients of nodes in the rainfall and wind speed layers increased.

In the clustering analysis results (Figure 15), there was a significant increase in the clustering coefficient of nodes in the rainfall and wind speed layers. The average coefficients were almost four times larger than before. However, there was no change in the nodes of the aircraft layer. 

Lastly, in the centrality analysis result, except for the KPO node in the aircraft layer, all nodes had different ranks in the new network. All nodes in the aircraft layer show a decrease, while most nodes in the rainfall and wind speed layers show an increase. The most dramatic change was observed in CJJ in the rainfall layer, which shows an increase of 22 ranks (23->1). Regarding the top 10 nodes ranked by centrality, the original network included nodes from all layers, whereas the new network only included nodes from the rainfall layer. Additionally, high-ranking nodes in the new network did not hold any significant meaning regarding the aircraft cancellation phenomenon. The analysis of the new network confirmed that weather factors and their relationships were more prominent compared with the original network, and the aircraft element had limited significance. In other words, this result can be seen as a network analysis of the relationship between rainfall and wind speed, rather than the relationship between weather and aircraft investigated in this study. Therefore, it is important to define links properly according to the characteristics of the target or phenomenon and research purpose when applying the MCN method.

In Section 3.2, we confirmed that the rainfall network contained hub nodes in the multilayer complex network. However, the number of aircraft cancelations due to wind speed was much higher compared with those due to rainfall. Why did the analysis result show that rainfall played such a significant role? To investigate the reason for this, we checked the structural characteristics of each climate network. By comparing the inter-layer links from each weather network to the aircraft, it can be observed that all the nodes in the network of wind speed were linked to the aircraft, whereas in the network of rainfall, links were absent only in the case of the MWX and YNY nodes. Regarding the total weight of the inter-layer links, the weight of the wind speed network was 12 times greater in comparison with that of the rainfall network. This was because the inter-layer links were calculated based on the data for aircraft cancellations. However, the intra-layer links show different results. In terms of the degree and strength of the inter-layer links, the rainfall network was overwhelmingly large with 182 links (rainfall) and 78 links (wind speed) and strengths of 62.04 (rainfall) and 14.70 (wind speed). The difference was obtained from the characteristics of each weather event. In the case of rainfall, the same rainfall event was observed in multiple regions. Therefore, the same rainfall phenomenon was shared at different observation points, and a high correlation between the sites became evident as the data from observation accumulated. However, in the case of wind, the frequency of occurrence and regional variations were more severe owing to the topography and geography [46]. The characteristics of the area had a greater influence on the wind speed than on rainfall, hence the disparity in their impacts. When interpreting the cancellation of aircraft by weather using these characteristics, we interpret the wind as behaving like a sudden event that occurred unexpectedly in different regions rather than occurring evenly across several regions. However, rainfall can affect multiple locations simultaneously, resulting in multiple cancellation events. Therefore, we could conclude that rainfall had a greater influence on aircraft operation than wind because it generated simultaneous events at multiple locations, unlike sudden and isolated events caused by wind. 

Despite the inclusion of more than 17 weather phenomena in aeronautical meteorological warnings, this study focused only on rainfall and wind speed as weather factors due to data availability. Rainfall and wind speed have specific quantitative criteria, such as 30 mm/h and 25 kt. Even though yellow dust and heavy snowfall also meet this criterion, ASOS stations do not record relevant weather data for them. Therefore, weather factors other than rainfall and wind speed could not be considered in this study. In addition to the 17 weather events, other factors, such as dust and mist, which can significantly affect cancellations, also have a great influence by reducing the range of sight. In future research, if additional weather layers are included in the network and spatial coverage is expanded, the analysis of aircraft cancellations due to weather in the global airline network can yield valuable insights. 

We applied the MCN method to analyze aircraft cancellations due to weather. Using the MCN method, we expressed the phenomena as the MCN, which simplified the analysis process. In comparison with analyzing a single-layer network using a complex network method, the multilayer complex network allowed for the integration of various elements into a unified system, enabling simultaneous consideration of their relationships. However, building the MCN correctly is crucial when applying the method. If researchers do not consider the characteristics of the target or phenomena and objectives of the study when creating the network, the method can lead to incorrect results. Therefore, a thorough understanding of the objectives should precede the application of the network.

## 5. Conclusions

In this study, aircraft cancellations due to weather were analyzed through the application of the MCN method. The MCN comprised three single-layer networks (rainfall, wind speed, and aircraft) and the inter-layer links were defined by the number of aircraft cancellations due to rainfall and wind speed. We applied several network analysis methods to examine the characteristics and performed centrality analysis based on adjacency information entropy. The results showed that the MCN contained all the characteristics of three single networks and inter-layer links also impacted the characteristics of the MCN. In terms of the network structure, it had high stability with hub nodes concentrated in the rainfall layer. In the centrality analysis result, CJU was identified as the most significant node in the network, as it held the highest importance from both social and meteorological perspectives. By applying inter-layer links between the rainfall and wind speed layers, we confirmed that it is crucial to create a network with a proper understanding of the target and objectives of the study. In the Republic of Korea, rainfall had a greater impact on aircraft cancellation than wind speed due to the characteristics of rainfall. Unlike previous aircraft studies using complex network analysis, this study considered weather factors in conjunction with aircraft and extracted useful information regarding aircraft cancellations in the Republic of Korea. Through the results, the study showed the possibility of creating an MCN with different kinds of data and it led to the expansion of the applicability range of the MCN. However, this study only applied rainfall and wind speed among the 17 weather factors in aeronautical meteorological warnings. Additionally, the research area was limited to domestic air routes. Therefore, we can gain deeper insights into aircraft cancellation by adding additional weather factors and expanding the research area to include international air routes. It will be helpful to understand and establish alternatives for aircraft cancellations. 

## Figures and Tables

**Table 1:** Criteria of aeronautical meteorological warnings according to the types of weather [34].

Type	Criteria
Tropical cyclone	Strong winds or heavy rainfall due to tropical cyclones are expected to reach warning levels.
Thunder and lighting	Thunder and lightning occur or are expected at the airport.
Heavy snowfall	Snowfall occurs or is expected to be more than 3 cm/24 h.
Gust	Gale (10 min mean surface wind speed with 25 kt or more, or gusts with 35 kt or more) occurs or is expected.
Ceiling	A ceiling occurs or is expected to be at a level below a criterion agreed upon by the local meteorological authority, air traffic services authority, and aircraft operations at the aerodrome.
Heavy rainfall	Rainfall occurs or is expected to be at 30 mm/h or more, or 50 mm/3 h or more.
Yellow dust	Yellow dust (1 h mean concentration of fine dust (${PM}_{10}$) with more than 400 μg/m^3^ or visibility less than 5000 m) occurs or is expected.
When the following phenomena are observed or predicted: (1) Hoar frost or rime, (2) freezing precipitation, (3) frost, (4) blowing sand or dust, (5) dust or sand storm, (6) squall, (7) volcanic ash, (8) hail, (9) volcanic ash deposit, and (10) toxic chemicals.	

In terms of cancellations, the decision to cancel depends on the aircraft route. Aircraft flying on medium- and long-distance routes are canceled when the phenomena included in the aeronautical meteorological warnings are predicted to make flying difficult for 1–2 days. On the other hand, the weather at the time of takeoff determines the cancellations in the case of short-distance or domestic routes. This study considered cancellations due to rainfall (heavy rainfall) and wind speed (gust) because both have clear quantitative criteria and nearby weather stations have relevant observation data.

**Table 2:** Number of events above the rainfall and wind speed aircraft cancellation criteria.

Airport	Rainfall (>30 mm/h)	Wind Speed (>25 kt)
KWJ	158	3
KUC	141	25
TAE	121	0
MWX	144	120
PUS	231	76
GMP	160	2
YNY	167	2
RSU	196	338
USN	146	2
WJU	140	0
CJU	169	50
HIN	214	0
CJJ	149	0
KPO	137	4

**Table 3:** Results of the centrality analysis: Top 5 nodes in each single layer network are represented by italicized and bold letters. In the rainfall and wind speed networks, KUV was the most important node. On the other hand, CJU was the most influential node in the aircraft network.

Rainfall			Wind Speed			Aircraft		
Node	Adjacency Information Entropy	Rank	Node	Adjacency Information Entropy	Rank	Node	Adjacency Information Entropy	Rank
***KWJ***	***3.605***	***5***	KWJ	2.664	6	KWJ	***1.083***	***4***
***KUV***	***3.626***	***1***	***KUV***	***3.094***	***1***	KUV	0.000	12
***TAE***	***3.607***	***4***	TAE	0.000	11	TAE	0.148	11
MWX	3.584	9	***MWX***	***3.012***	***4***	MWX	0.766	7
PUS	3.591	7	***PUS***	***3.068***	***2***	PUS	***1.059***	***5***
GMP	3.557	12	GMP	2.455	7	***GMP***	***1.587***	***3***
***YNY***	***3.615***	***2***	YNY	2.282	10	***YNY***	***1.660***	***2***
RSU	3.581	11	***RSU***	***2.853***	***5***	RSU	0.728	8
USN	3.583	10	USN	2.389	9	USN	0.594	10
WJU	3.546	14	WJU	0.000	11	WJU	0.000	12
CJU	3.552	13	***CJU***	***3.048***	***3***	***CJU***	***2.002***	***1***
HIN	3.604	6	HIN	0.000	11	HIN	1.036	6
***CJJ***	***3.614***	***3***	CJJ	0.000	11	CJJ	0.000	12
KPO	3.586	8	KPO	2.439	8	KPO	0.7226	9

**Table 4:** Results of the centrality analysis: Top 5 nodes in the MCN are represented by italicized and bold letters. On average, the rainfall layer had the highest rank among the layers. As for the rank of each node, the CJU node in the aircraft network had the highest entropy.

Layer	Node	Adjacency Information Entropy	Rank
Rainfall	KWJ	3.701	9
	KUV	1.872	26
	TAE	2.766	20
	MWX	3.728	7
	PUS	3.274	12
	GMP	3.696	10
	YNY	2.879	18
	RSU	3.062	16
	***USN***	***3.969***	***5***
	***WJU***	***4.544***	***3***
	CJU	2.853	19
	HIN	2.357	25
	CJJ	2.475	23
	KPO	2.735	21
Wind speed	KWJ	3.115	14
	KUV	0.074	42
	TAE	0.527	35
	***MWX***	***4.152***	***4***
	PUS	3.708	8
	GMP	3.070	15
	YNY	2.970	17
	RSU	3.641	11
	USN	2.359	24
	WJU	0.377	38
	CJU	3.876	6
	HIN	0.514	36
	CJJ	0.413	37
	KPO	2.511	22
Aircraft	KWJ	1.561	27
	KUV	0.285	39
	TAE	1.033	33
	MWX	1.533	29
	PUS	1.033	34
	***GMP***	***4.842***	***2***
	YNY	3.173	13
	RSU	1.145	30
	USN	1.533	28
	WJU	0.285	40
	***CJU***	***4.890***	***1***
	HIN	1.145	31
	CJJ	0.285	41
	KPO	1.145	32

**Table 5:** Correlation between rainfall (≥30 mm/h) and wind speed (≥25 kt); CJU and KPO only had significant correlation values.

Node	Correlation
KWJ	0.078
KUV	0.001
MWX	0.129
PUS	0.150
GMP	0.024
YNY	−0.003
RSU	0.191
USN	0.007
CJU	0.357
KPO	0.317

We also calculated the event synchronization to check the degree of synchronization between the rainfall and wind speed. The synchronization results show that all values were less than 0.1. Our analysis of the correlation and synchronization results led us to conclude that there was no significant relationship between rainfall and wind speed. Therefore, we did not include the inter-layer links between the different layers in the multilayer complex network.

## Data Availability

Daily rainfall and wind speed data are available on the Open MET Data Portal (https://data.kma.go.kr, accessed on 19 June 2023) managed by the Korea Meteorological Administration. Flight schedules and flight cancellation data are available on the Air Portal (https://www.airportal.go.kr, accessed on 19 June 2023), which is managed by the Ministry of Land, Infrastructure, and Transport, Republic of Korea.
